# A Fatal Sepsis Caused by Hyaluronate Knee Injection: How Much the Medical History and the Informed Consent Might Be Important?

**DOI:** 10.1155/2017/1518401

**Published:** 2017-02-23

**Authors:** F. Manfreda, G. Rinonapoli, A. Nardi, P. Antinolfi, A. Caraffa

**Affiliations:** ^1^Department of Orthopedics and Traumatology, University of Perugia, Perugia, Italy; ^2^Division of Orthopedics and Trauma Surgery, Santa Maria della Misericordia Hospital, Perugia, Italy

## Abstract

The incidence of Osteoarthritis (OA) is gradually increasing worldwide due to two main reasons: longer life expectation and increased functional demand. Several treatment options have been proposed for this disease. Conservative treatment has the goal to improve the quality of life, reduce pain, and prevent the progression of the disease. Hyaluronate viscosupplementation is one of the most used infiltrative treatments for OA, but, despite its common use, clinical efficacy is still under question. Though adverse reactions for this medical option are actually rare, septic arthritis is a very scaring complication. We present a case report of a 59-year-old man who has been submitted to only one knee hyaluronate injection and consequently reported a severe septic arthritis and systemic sepsis, which lead to the death of the patient. We recommend producing correct guidelines for a clean aseptic procedure of injection to obtain proper consensus from the patient and to pay attention to his clinical history and comorbidities before acting any kind of invasive treatment, including joint injection.

## 1. Introduction

Knee Osteoarthritis (OA) is one of the commonest orthopedic pathologies [1] whose prevalence is increasing worldwide. This burden will continue to increase in the general population [2]; actually, it gives functional deficits in 10% of individuals over the age of 55 years [3]. Several treatments for OA have been proposed during the years in a very large range of strategies [4], including interventions to increase tolerance for functional activity, improve quality of life, prevent the disease, and stop its progression [5]. Among them, intra-articular injection is one of the most used treatments in order to reduce symptoms, both in early and in the severe stages of the disease [6].

Viscosupplementation with intra-articular hyaluronic acid was approved as a conservative option for OA in 1997 by FDA (Food and Drug Administration) [7, 8]. Important benefits for this kind of treatment have been proven in the last years: a recent Cochrane review found overall benefits of viscosupplementation in comparison to placebo for pain, function, and patient global assessment scores [9]. But there are also some other clinical trials and reviews that report fewer or inconsistent beneficial effects [10, 11].

In general, an excellent safety profile for this treatment method has been widely shown, with few common adverse events, such as mild injection site pain and swelling [12–16]. However, there are several adverse effects that may occur in either poor injection technique or patients with preexisting morbidities [17, 18]. While periarticular complications are the most common, with an incidence until 43%, intraarticular adverse reacts (2%–10%) and in particular infections (0,001%–0,072%) are the most dangerous [19].

In this report, we describe a case of a man who received an intra-articular hyaluronate injection for knee OA and developed a severe articular infection and systemic sepsis.

## 2. Case Presentation

The patient is a 59-year-old male affected by bilateral knee OA. He has already been subjected to surgical intervention of total hip arthroplasty for Hip OA. Past medical features included a severe obesity, with a body mass index higher than 32, chronic bronchial asthma treated with steroid drugs, and systemic arterial hypertension treated with *β*-blockers. VAS score corresponded to 8-9.

Knee X-rays had shown specific characters of articular OA that we could categorize as grade 3 of disease according to Kellgren-Lawrence scale [20].

His right knee pain was difficult to control with conservative measures, including NSAIDs and narcotics. Oral steroid drugs were improved by his family physician in order to get pain relief but with no benefits.

So he was submitted to an intra-articular hyaluronate injection, without immediate complications. A high molecular weight hyaluronic acid has been used (about 1200 kDa). Cleaning technique was employed prior to the treatment, including the use of antiseptic solution and sterile gloves; sterile infiltrative practice in clean condition has been conducted.

About 48 hours after that, he reported a severe fever, at about 102.2 Fahrenheit/39° Celsius. Antipyretic drugs did not decrease body temperature and in a few hours his general clinical conditions got worse.

72 hours after the injection, he was hospitalized. The input diagnosis was septic shock, which was quickly treated with adequate antibiotic and support therapy. CRP and ESR values reported a gradual reduction and the shock had a quick remission.

Blood and knee synovial fluid cultures had clear and dramatic positive results for two different atypical microorganisms: multiresistant* Escherichia Coli* and multiresistant* Klebsiella*.

Specific antibiogram had shown sensitivity to very few antibiotics for this kind of bacteria. A combination of Vancomycin (500 mg I.V., 4 times a day) and Cephalosporin (Ceftriaxone 2 gr I.V., twice daily) was used for the full treatment.

**Figure 1:**
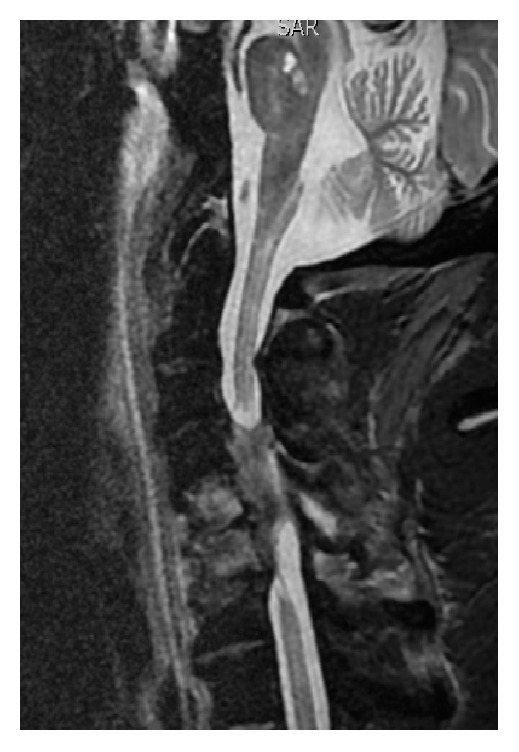
Septic involvement of cervical spine and spinal cord.

Anyway, twelve days after admission, the patient presented a complete flaccid paralysis. An encephalic and spinal MRI was performed, showing a septic involvement of more than two vertebrae, in particular from C5 to C7, and the corresponding cervical spinal cord (Figure 1).

The patient was mainly assisted by the Spinal Unit of our hospital which cooperated with the orthopedic team.

Neurologists performed a proper evaluation of spinal function by the ASIA Standard Neurological Classification of Spinal Cord Injury [21]: results were inauspicious. In fact, he did not report any positive response to stimulation and paralysis was complete and permanent.

He has been treated with an intensive rehabilitation program for several weeks.

**Figure 2:**
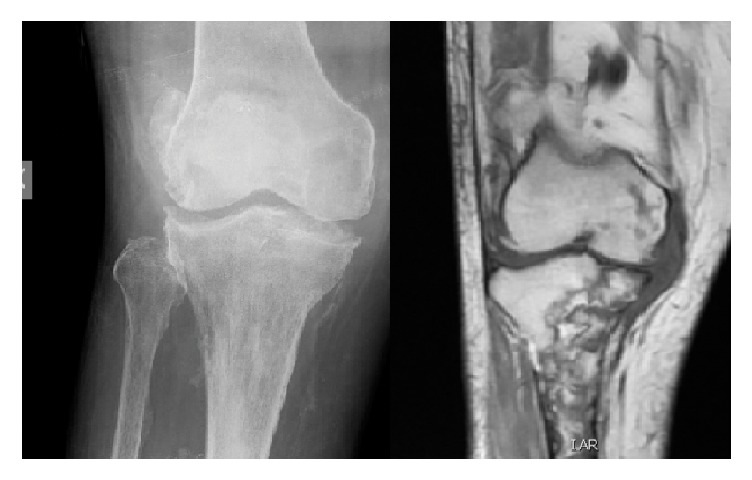
X-ray and MRI showing both knee septic arthritis and tibial and femur osteomyelitis.

Meanwhile, he presented a fistula in the proximal region of the leg. So, a new knee X-ray and an MRI were conducted: a severe septic arthritis of the knee and osteomyelitis of tibia and femur were confirmed (Figure 2).

Surgical debridement of the knee and the leg was performed in order to reduce their septic involvement.

**Figure 3:**
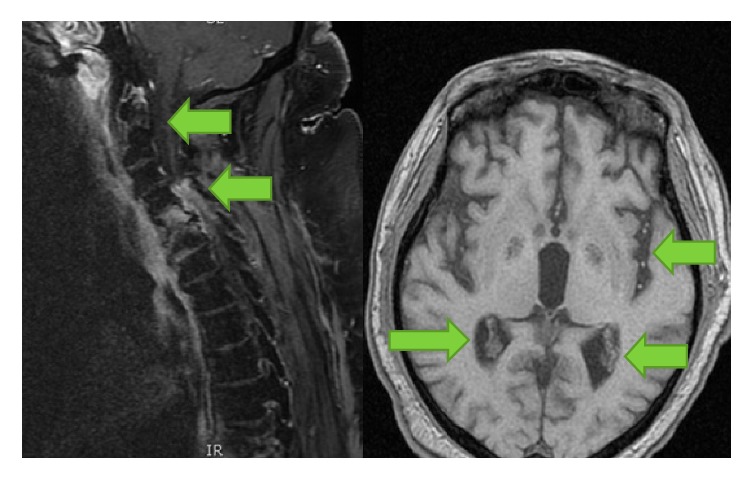
Cephalic and cervical MRI showing septic collections in cerebral ventricles and cervical spinal cord.

Despite his rehab program, the patient did not report any neurological improvements; at a 5-week follow-up, a new cephalic and cervical MRI showed worse conditions: septic features have been found in cerebral ventricles, and spinal disease became larger than before: almost the full cervical spine (C2–C7) was involved by the infection (Figure 3).

The higher spinal cord involvement has made self-contained breathing impossible. Thus, the patient started artificial breathing.

At about four months from the entrance to the hospital, breath complications arose, which led to a poor prognosis. The patient died because of severe acquired pneumonia, caused by* Pseudomonas aeruginosa*.

## 3. Discussion

As mentioned, sodium hyaluronate injection has been proven to be quite safe, with low incidence of adverse reaction [16, 22]. Septic arthritis is a rare but potentially fatal [8, 23–25] severe complication after an intra-articular injection. Although injective practice is conducted in sterile ways, the incidence of joint infection is estimated to be 0,01%–0,072% [19]. In scientific literature, there are several reports about infections for this kind of treatment [8, 16–26]. The causative bacteria could be aerobic, anaerobic, or mixed flora;* S. Aureus* is the most common [27]. Risk factors for joint infections include immunosuppression, diabetes, trauma, and operative infections. Sodium hyaluronate injection is contraindicated if skin disease or current infection is present at the injection site, but there is no mention of contraindication for viscosupplementation in immunosuppressed patients or patients with underlying malignancy [8, 28].

Our patient was used to take steroid drugs for his chronic asthma, and there is strong evidence about a connection between chronic steroid therapy with both immunosuppression and increase of atypical infection [29, 30]. He was affected by two uncommon bacteria for joints that presented resistance to several antibiotics, with poor sensibility to Vancomycin and Cephalosporin. Antibiotic resistance has caused a poor response to therapy. So we could think that chronic steroids, which had been increased to relieve pain, could have caused an atypical infection. This could have been also the cause of rapid spreading of infection from joint to bloodstream.

Then, there is strong evidence about increased risk of infection in case of obesity [31, 32]; our patient was not used to practicing any sport activity and he had a very high BMI.

Actually, we do not know exactly how bacteria have been inoculated in his joint. Maybe, even if skin was safe, it might have been the source of inoculation in this immunodepressive condition.

Clinical history of patients should be accurately examined in order to decide proper treatment options related to comorbidities and to evaluate risks of complications related to invasive action.

Another important issue concerns informed consensus. American College of Rheumatology and Italian Consensus of Rheumatology agree about the need of obtaining clear consensus by patients who will be submitted to a joint injection or an arthrocentesis [33, 34]. It should be acquired both orally and in writing, generally after explaining what are the risks and benefits of such action.

We think that a proper and accurate evaluation of the clinical history of patients and the acquirement of clear consensus are essential for every kind of medical action towards patients. Joint injection, too, should indeed be considered an invasive treatment.

Clean careful practice is recommended by using sterile gloves, sterile materials, and clean procedure. Then, it should be noted that there are no international common agreements about guidelines for implementation of clean and aseptic technique [8].

Apart from complications, the debate about long-term effectiveness of intra-articular hyaluronate for OA is always open [9, 11, 35–38].

Several studies show overall benefits for this kind of treatment: it could be considered a viable option in younger patients with less severe disease [9, 35, 36], though some other systematic reviews did not succeed in proving efficacy of hyaluronate joint injection for regression of articular damage [11, 37, 38].

## 4. Conclusion

The overall incidence of side effects of hyaluronate acid intra-articular injection is lower than 3% [19]. Even if adverse events are quite rare, septic arthritis could be a life-threatening complication. We have presented a case report that underlines the importance of the accurate evaluation of clinical history and comorbidities and the need of clear informed consensus for every invasive treatment, including joint injection. Furthermore, we recommend clear internationally accepted guidelines for clean methods regarding intra-articular injections.
